# Malignant Adenomyoepithelioma of the Breast with Lymph Node Metastasis: A Detailed Immunohistochemical Study

**DOI:** 10.1155/2012/305858

**Published:** 2012-01-18

**Authors:** Ahlam A. Awamleh, Mihir Gudi, Sami Shousha

**Affiliations:** Department of Histopathology, Charing Cross Hospital, Imperial College Healthcare NHS Trust and Imperial College, Fulham Palace Road, London W6 8RF, UK

## Abstract

Malignant adenomyoepithelioma of the breast is a rare tumour with around 30 cases reported in the literature. Metastases associated with these tumours are usually haematogenous. Axillary lymph node metastases are thought to be unusual, and it has been recently suggested that axillary node dissection is not indicated unless clinically palpable. We here present a case of a 63-year-old woman, who developed a malignant adenomyoepithelioma with axillary lymph node metastasis, that included epithelial and myoepithelial elements, in spite of the absence of clinically enlarged nodes. We suggest that histological examination of axillary sentinel node(s) or node sampling may be worthwhile in this condition.

Adenomyoepithelioma of the breast is a benign neoplasm characterised by biphasic proliferation of epithelial and myoepithelial cells [1]. It resembles adenomyoepithelioma of salivary glands as first described by Hamperl [2]. Malignant change can occur rarely in one or both cellular components [3] and can be either a pure myoepithelial carcinoma or a combined malignant adenomyoepithelioma [1]. Around 30 cases of malignant adenomyoepithelioma have been reported in the literature [1, 3–5].

A 63-year-old woman presented with a mass in the left breast. A core biopsy showed intraductal papilloma with atypical hyperplasia (B3). This was removed by wide local excision. Grossly, the biopsy included two small greyish white soft nodules, each measuring 1 cm in diameter. Microscopic examination showed multiple intraductal papillary lesions. In some areas, the papillae were covered by a single layer of epithelial cells with underlying several layers of myoepithelial cells which stained positively for smooth muscle actin, p63, and CD10, appearances which were interpreted as adenomyoepithelioma (Figure 1). The epithelial cells were ER negative and many were CK5 and 14 positive, indicating that they are of basal-like rather than luminal type. Other areas of the lesion consisted of solid proliferation of a mixture of these epithelial and myoepithelial cells and showed abundant mitotic figures and marked nuclear pleomorphism (Figure 2(a)), with evidence of peripheral invasion (Figure 2(b)). The features were interpreted as a malignant adenomyoepithelioma developing in continuity with a benign adenomyoepithelioma. The lesions reached the excision margins and reexcision was recommended.

A mastectomy was carried out with axillary lymph node sampling. Pathological examination showed a partly cystic and partly solid tumour measuring 7 × 4 × 4.5 cm, that showed similar features to those seen previously and consisted of a mixture of epithelial and myoepithelial cells arranged in a benign adenomyoepithelioma fashion in some areas, which merged with areas showing malignant features as those described above with invasion of adjacent breast tissue. One of the two dissected lymph nodes showed a 1.8 mm metastatic focus which was positive for CK8/18, CK19, AE1/AE3, CK5/6, CK14, SMA, and CD10, indicating the presence of epithelial and myoepithelial elements (Figure 3).

Malignant adenomyoepithelioma is generally heralded by a long history of a stable breast mass followed by rapid growth phase [1]. Grossly, the tumour is usually nodular and the nodules may show cystic changes as well as necrosis and foci of calcification. Histological features of malignant transformation include nuclear atypia, increased mitotic activity, necrosis, and infiltrative growth pattern [3, 4]. In our case, all these features were present, except for necrosis, and the dual cellular nature was established by immunohistochemistry in the primary lesion and lymph node metastasis.

 Malignant adenomyoepithelioma has the potential for distant metastases. These typically occur in lesions larger than 2 cm [3] and in those with high-grade malignant component [1]. Distal metastasis were described in 8 (32%) out of 25 cases reviewed by two authors [1, 3], involving lungs, brain, and soft tissues. Other authors described metastasis in the liver, bone, thyroid gland, and mediastinal lymph nodes [6–8].

Axillary lymph node involvement is thought to be unusual, hence a recent review article has suggested that axillary node dissection is not indicated unless there is clinically involved lymph nodes [1]. However, metastases to axillary nodes have been reported in 2 previous cases of malignant adenomyoepithelioma [9, 10], in addition to the current case where no palpable lymph nodes were present. Thus, we would suggest that examination of sentinel node(s) or axillary node sampling may be worthwhile in this condition.

## Figures and Tables

**Figure 1:**
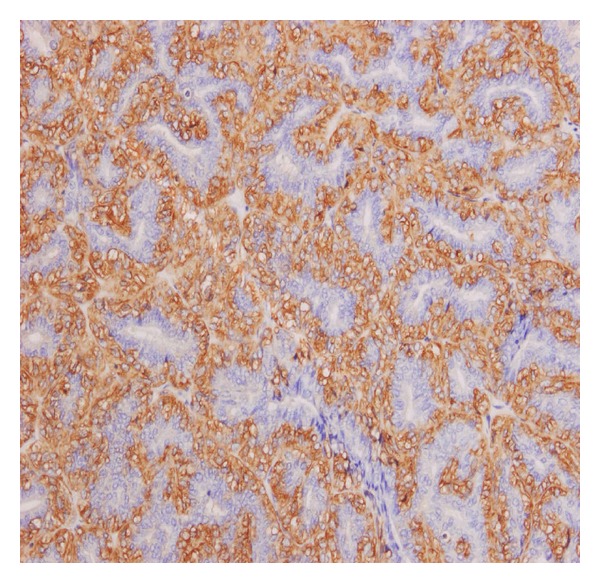
Benign adenomyoepithelioma part of the lesion stained with smooth muscle actin to show the myoepithelial component stained brown.

**Figure 2:**
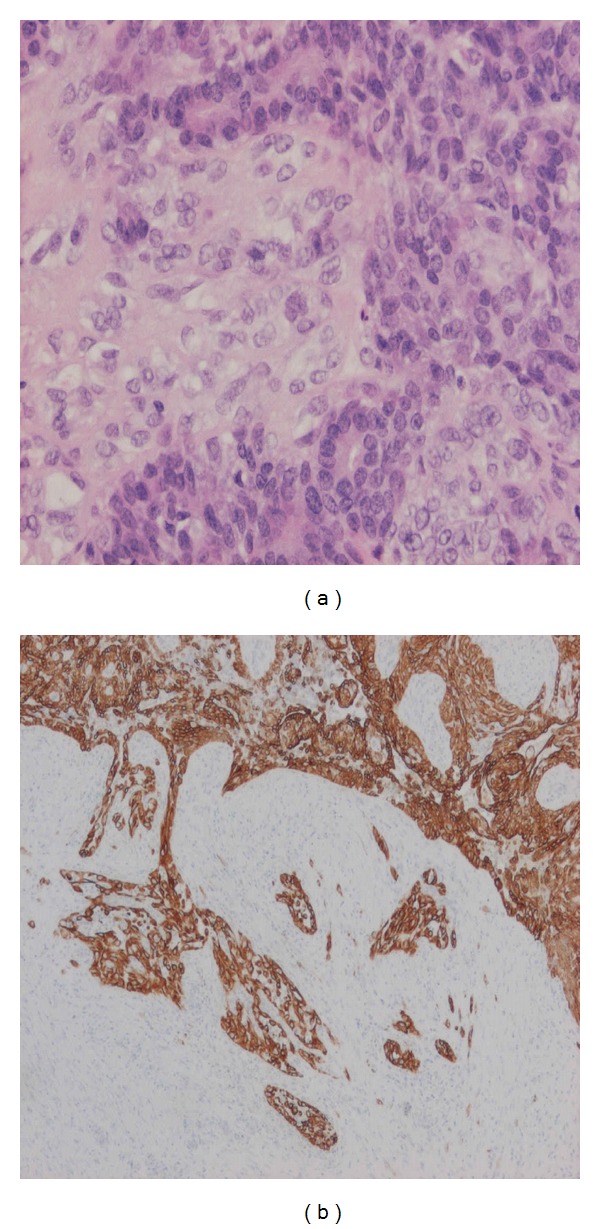
(a) Malignant component: solid area stained with H&E showing dual-cell population with marked nuclear pleomorphism. (b) Malignant component: invasive edge of the lesion stained with cytokeratin 5.

**Figure 3:**
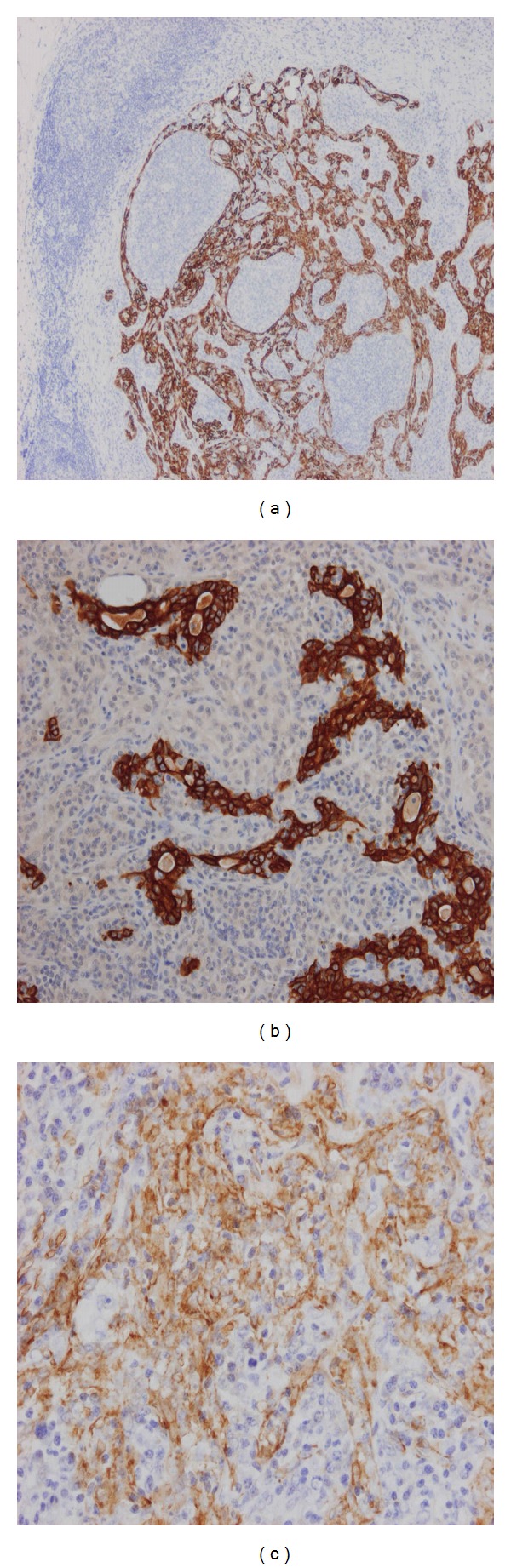
(a) Lymph node metastasis: CK5 staining both epithelial and myoepithelial elements. (b) Lymph node metastasis: CK19 staining epithelial element. (c) Lymph node metastasis: smooth muscle actin staining myoepithelial element.
